# Risk of mortality during and after the 2011 Great East Japan Earthquake and Tsunami among older coastal residents

**DOI:** 10.1038/s41598-017-16636-3

**Published:** 2017-11-29

**Authors:** Jun Aida, Hiroyuki Hikichi, Yusuke Matsuyama, Yukihiro Sato, Toru Tsuboya, Takahiro Tabuchi, Shihoko Koyama, S. V. Subramanian, Katsunori Kondo, Ken Osaka, Ichiro Kawachi

**Affiliations:** 1 Tohoku University Graduate School of Dentistry, Center for Epidemiology, Biostatistics and Clinical Research, 4-1 Seiryo-machi, Aoba-ku, Sendai, Miyagi 980-8575 Japan; 2 Tohoku University Graduate School of Dentistry, Department of International and Community Oral Health, 4-1 Seiryo-machi, Aoba-ku, Sendai, Miyagi 980-8575 Japan; 3 Harvard T.H. Chan School of Public Health, Department of Social and Behavioral Sciences, 677 Huntington Avenue, Boston, Massachusetts 02115 USA; 4 Cancer Control Center, Osaka International Cancer Institute, 1-69, Ohtemae 3-Chome, Chuo-ku,, Osaka 541-8567 Japan; 5 Chiba University, Center for Preventive Medical Sciences, 1-8-1, Inohana, Chuo-ku, Chiba-shi, Chiba 260-8670 Japan; 6 Center for Gerontology and Social Science, National Center for Geriatrics and Gerontology,, 7-430 Morioka-cho, Obu, Aichi 474-8511 Japan; 7 Tohoku University, International Research Institute for Disaster, 468-1, Aramaki, Aoba-ku, Sendai, Miyagi 980-0845 Japan

## Abstract

The Japan Gerontological Evaluation Study is a nationwide cohort study of individuals aged 65 years and older established in July 2010. Seven months later, one of the study field sites was directly in the line of the 2011 Great East Japan Earthquake and Tsunami. Despite the 1-hour warning interval between the earthquake and tsunami, many coastal residents lost their lives. We analyzed the risk of all-cause mortality on the day of the disaster as well as in the 38-month interval after the disaster. Among 860 participants, 33 (3.8%) died directly because of the tsunami and an additional 95 people died during the 38-month follow-up period. Individuals with depressive symptoms had elevated risk of mortality on the day of the disaster (odds ratio = 3.90 [95% CI: 1.13, 13.47]). More socially connected people also suffered increased risk of mortality, although these estimates were not statistically significant. In contrast, after the disaster, frequent social interactions reverted back to predicting improved survival (hazard ratio = 0.46 (95% CI: 0.26, 0.82)). Depressive symptoms and stronger social connectedness were associated with increased risk of mortality on the day of the disaster. After the disaster, social interactions were linked to improved survival.

## Introduction

An estimated 99,700 people have lost their lives annually on average due to global natural disasters in the past decade^1^. In addition to direct impact, disasters also increase mortality as a result of acute stress, injuries, disruptions in medical care, heart disease, suicide, and other causes^2–10^. Many victims develop posttraumatic stress disorder or other mental disorders^11–14^. Acute stress after a disaster is thought to trigger cardiovascular events^4,5,10^. Victims who are forced to relocate frequently experience worse living conditions as well as disruption of their social networks, and these factors may also adversely affect mental and physical health^2,13^.

Although a framework for disaster risk reduction was developed by the United Nations Office in 2015^15^, potential risk factors for mortality pre-dating the events are not well understood. An obvious reason is that most studies of disasters are conducted after the fact, and they must rely on retrospective recall among the survivors, limited medical records, or ecological data^2–9,16–21^. A notable exception is the Study of the Tsunami Aftermath and Recovery (STAR) conducted before and after the 2004 Indian Ocean Tsunami that killed more than 130,000 people^22^. However, because their baseline survey was focused on social and economic conditions, information on health and behavioral risk factors was not collected. Other exceptions to the absence of pre-disaster information include cohort studies from New Zealand and Sweden that were able to take advantage of the fact that disaster struck in the midst of ongoing follow-up^23,24^; however, these studies did not examine mortality as an outcome.

Despite the about 1-hour warning interval between the earthquake and tsunami triggered by the 2011 Great East Japan Earthquake and Tsunami, 15,894 people lost their lives and 2,546 people remained missing^25^. The maximum run-up height of the tsunami recorded 40.5 m, and 90% of the deaths were due to drowning^26^. Pro-social behavior, such as helping others to evacuate, may have increased the risk of mortality during the disaster^27–29^, but epidemiological evidence remains scarce. In addition, more than 60% of fatalities were older adults^18,26^. Although older people are considered to be a vulnerable group in disaster planning policy^30–32^, specific risk and protective factors for mortality remain poorly understood. For example, depression is quite prevalent in the older population^33^, and possibly contributes to delayed evacuation in the event of disaster. In the present analysis, we conducted a longitudinal assessment of risk factors for tsunami-related mortality using baseline data collected 7 months before the 2011 earthquake and tsunami.

## Results

**Figure 1:**
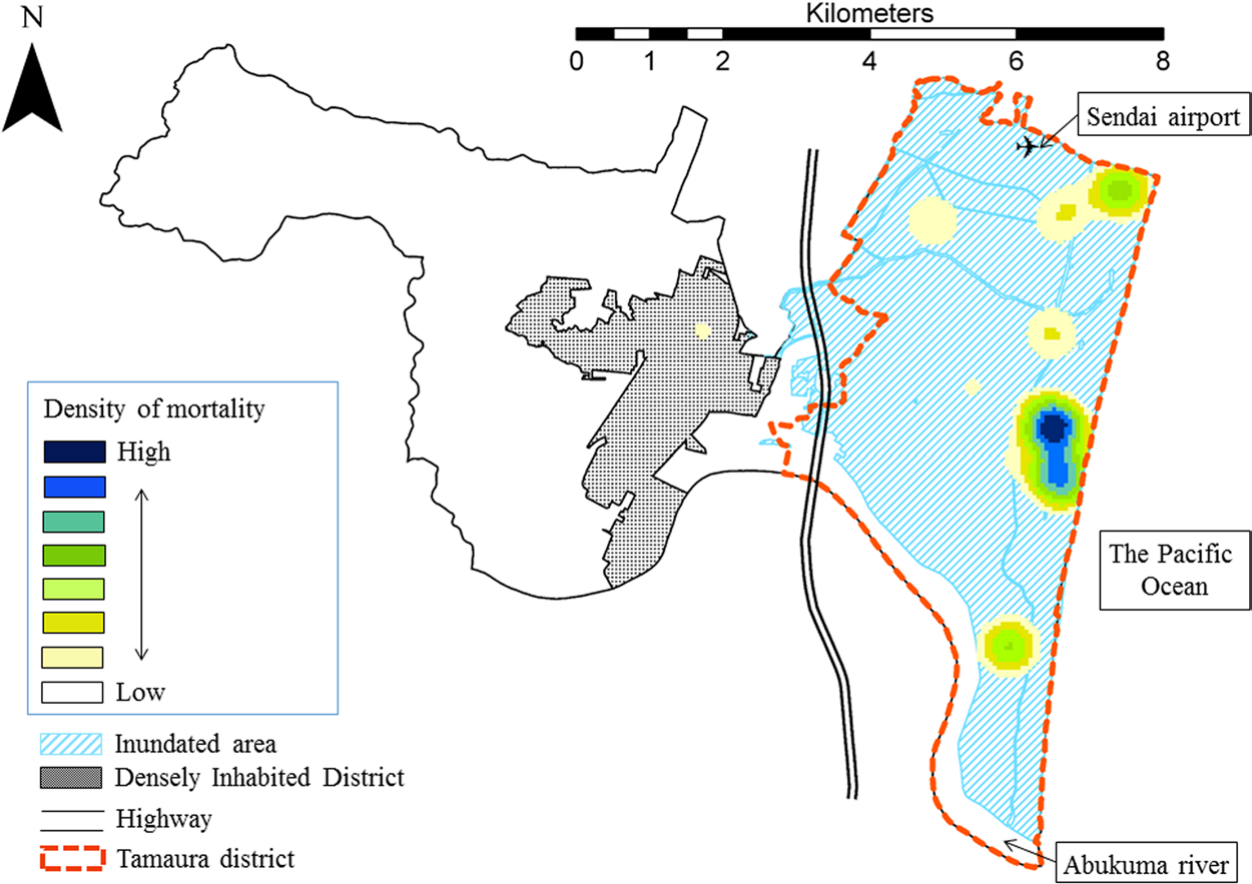
A heat map of Iwanuma City, the present study field, showing the density of mortality on the day of the Great East Japan Earthquake and Tsunami and the tsunami inundated area (N = 4,873 on March 11, 2011). The area surrounded by red dot-line and inundated by the tsunami is Tamaura district. Respondents living in the Tamaura district (N = 860) were included into the current analysis. The southern part of Tamaura district shown in the white color is the Abukuma River. Hiroyuki Hikichi edited the geographic data using ArcGIS Pro 1.1. (Esri, Redlands, California, USA), which were obtained from City Bureau (http://fukkou.csis.u-tokyo.ac.jp) and National Spatial Planning and Regional Policy Bureau (http://nlftp.mlit.go.jp/ksj/), Ministry of Land, Infrastructure, Transport and Tourism.

**Figure 2:**
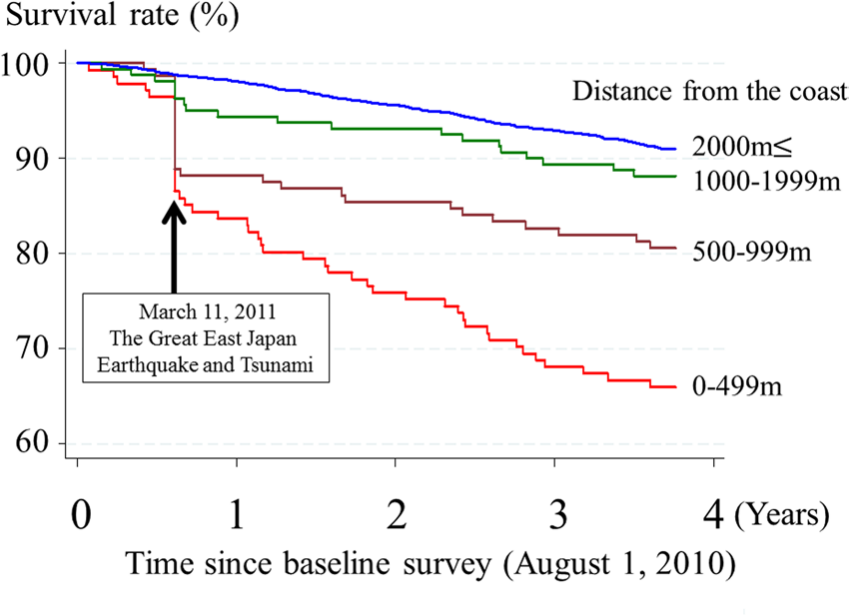
Survival rate (%) by distance living from the coast since the baseline survey for all districts in Iwanuma city (N = 4,937 on August 1, 2010).

**Figure 3:**
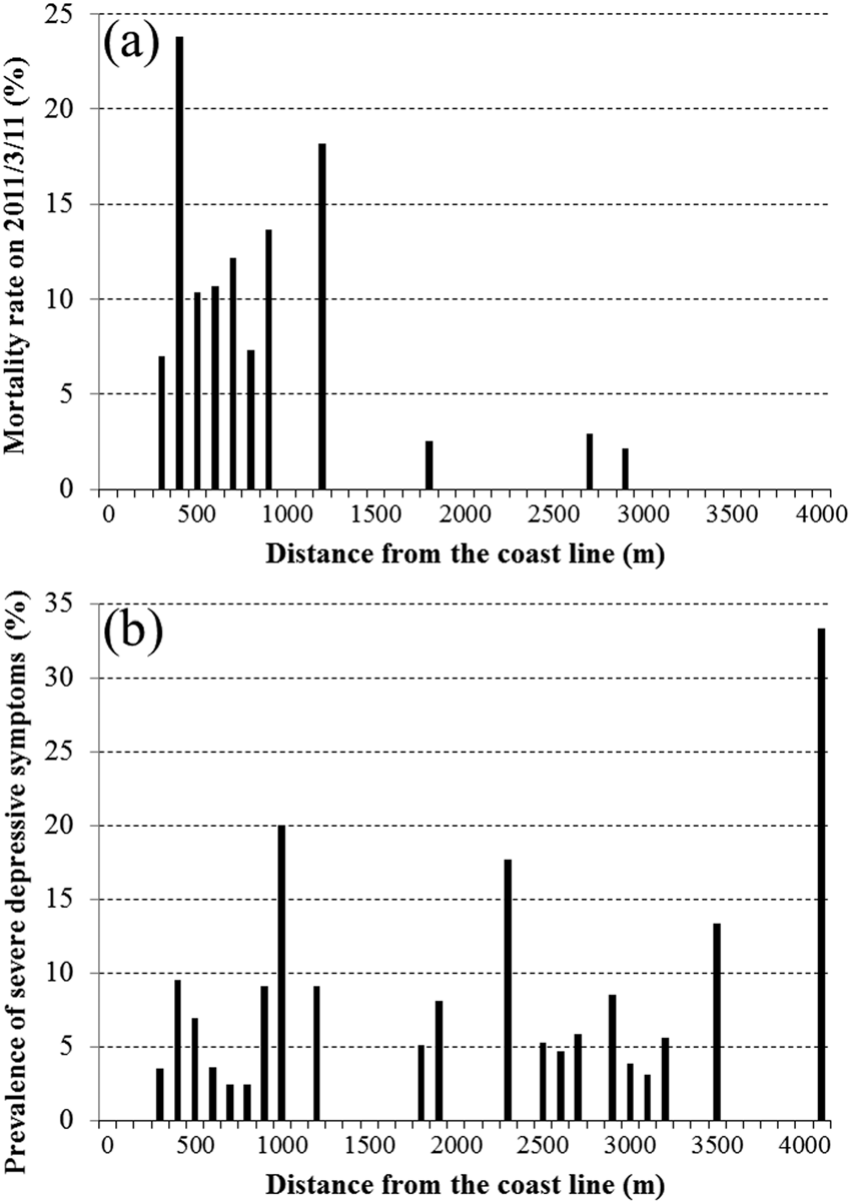
(**a**) Mortality rate (%) on the day of the Great East Japan Earthquake and Tsunami by distance living from the coast in Tamaura districts (N = 860). (**b**) Prevalence of the participants with severe depressive symptoms (%) before the day of the Great East Japan Earthquake and Tsunami by distance living from the coast in Tamaura districts (N = 860).

The mean age of participants was 75.6 (SD = 7.7) years. On the day of the disaster 33 participants died (mortality rate = 3.8%). An additional 95 participants died in the 3.15-year interval (mean follow-up period was 2.96 years) after the disaster. Baseline characteristics and mortality rates on the day of and day after the disaster are shown in Table 1. On the day of the disaster, those living less than 1 km from the coast (Figs 1, 2, and 3a), as well as those with pre-existing severe depressive symptoms had higher mortality rates. Participants co-habiting with others tended to have higher mortality than people living alone on the day of the disaster. However, after the disaster, people living alone experienced higher mortality. After the disaster, participants without any social interactions had higher mortality rates though it was not observed during the disaster.

**Table 1:** Baseline characteristics and mortality (%) owing to the Great East Japan Earthquake and Tsunami: data for the day of and after the disaster.

		On the day (Mar/11/2011, N = 860)	After Mar/12/2011 to May/5/2014 (N = 827)
		N (mortality %)	N (mortality %)
Distance from the coast	0–499 m	136 (10.3)	122 (23.8)
	500–999 m	142 (9.9)	128 (9.4)
	1000–1999 m	157 (1.9)	154 (8.4)
	2000–4100 m	425 (0.5)	423 (9.7)
Sex	Men	346 (5.5)	327 (12.5)
	Women	514 (2.7)	500 (10.8)
Age	65–69 years	238 (1.7)	234 (2.1)
	70–74 years	183 (3.3)	177 (7.9)
	75–79 years	175 (6.9)	163 (10.4)
	80–84 years	144 (2.8)	140 (12.9)
	≥85 years	120 (5.8)	113 (36.3)
Education	<10 years	487 (4.7)	464 (9.5)
	10–12 years	193 (2.1)	189 (12.7)
	> 12 years	82 (3.7)	79 (3.8)
Household	Living alone	68 (1.5)	67 (28.4)
	Co-habiting with others, but not parent(s)	618 (3.7)	595 (9.4)
	Living with parent(s)	59 (6.8)	55 (3.6)
Social interactions	Not meeting any friends	95 (3.2)	92 (37.0)
	Meeting some friends	686 (3.2)	664 (7.4)
Physical height	<150 cm	242 (3.7)	233 (12.9)
	150–159 cm	277 (3.2)	268 (8.2)
	≥160 cm	226 (3.5)	218 (9.2)
BMI	<18.5 kg/m^2^	40 (2.5)	39 (46.2)
	18.5–24.9 kg/m^2^	461 (3.3)	446 (8.5)
	≥25.0 kg/m^2^	236 (4.2)	226 (6.6)
Depressive symptoms	Normal	437 (3.0)	424 (7.3)
	Mild	161 (3.1)	156 (14.7)
	Moderate	69 (4.3)	66 (15.2)
	Severe	39 (12.8)	34 (26.5)
ADL	Independent	710 (3.9)	682 (7.0)
	Partially disabled	76 (3.9)	73 (30.1)
	Disabled	46 (2.2)	45 (51.1)
Comorbidity	Cancer; no	786 (3.8)	756 (11.0)
	Cancer; yes	38 (5.3)	36 (27.8)
	Heart diseases; no	682 (4.0)	655 (11.5)
	Heart diseases; yes	142 (3.5)	137 (13.1)
	Stroke; no	791 (3.8)	761 (11.4)
	Stroke; yes	33 (6.1)	31 (19.4)
	Respiratory diseases; no	796 (3.8)	766 (11.2)
	Respiratory diseases; yes	28 (7.1)	26 (26.9)
Smoking	Never	469 (3.2)	454 (11.9)
	Past	184 (6.0)	173 (12.1)
	Current	99 (5.1)	94 (9.6)
Alcohol	Non	549 (3.6)	529 (13.4)
	Quitted	43 (7.0)	40 (10.0)
	Drink	232 (3.9)	223 (6.7)
Exercise	<30 minutes	342 (4.1)	328 (14.0)
	30–59 minutes	230 (3.5)	222 (6.3)
	60–89 minutes	95 (3.2)	92 (7.6)
	≥90 minutes	99 (5.1)	94 (2.1)

Table 2 presents the main risk factors for mortality on the day of and the day after the disaster (Supplementary Tables 1 and 2 show the results of other variables, univariate analysis, and sensitivity analysis). On the day of the disaster, compared to residents living 2000 m or more from the coast, participants living 0–499 m (odds ratio [OR] = 22.66 (95% confidence interval [CI]: 5.78, 88.84)) and 500–999 m (OR = 16.88 (95% CI: 4.33, 65.84)) had elevated risks of mortality (Table 2a). However, after the disaster, there was no significant association between distance and mortality after controlling for other characteristics.

**Table 2:** Main risk factors of mortality on the day of and day after the Great East Japan Earthquake and Tsunami from logistic regression models (on the day, Mar/11/2011, N = 860) and Cox proportional hazard models (after the disaster, Mar/12/2011 to May/5/2014, N = 827)^*^.

		On the day of the disaster (95% confidence interval) Adjusted odds ratio**	After the disaster Adjusted hazard ratio (95% confidence interval)
**(a) Environmental factor**			
Distance from the coast	≥2000 m	1	1
	1000–1999 m	3.01 (0.56, 16.16)	0.83 (0.42, 1.65)
	500–999 m	16.88 (4.33, 65.84)	0.76 (0.38, 1.51)
	0–499 m	22.66 (5.78, 88.84)	0.84 (0.43, 1.68)
**(b) Physical factors**			
Height	≥160 cm	1	1
	150–159 cm	2.20 (0.67, 7.19)	1.06 (0.52, 2.17)
	<150 cm	2.98 (0.75, 11.84)	1.31 (0.57, 2.97)
Sex	Women	1	1
	Men	2.74 (0.77, 9.68)	3.33 (1.44, 7.73)
Age	65–69 years	1	1
	70–74 years	2.25 (0.60, 8.44)	3.53 (1.24, 10.03)
	75–79 years	5.29 (1.47, 19.04)	3.41 (1.19, 9.80)
	80–84 years	2.32 (0.54, 9.98)	4.65 (1.61, 13.46)
	≥85 years	5.41 (1.26, 23.16)	10.90 (3.82, 31.10)
**(c) Social connectedness**			
Household	Living alone	1	1
	Co-habiting with others, but not parent(s)	3.04 (0.47, 19.74)	1.05 (0.54, 2.06)
	Living with parent(s)	6.67 (0.83, 53.71)	0.45 (0.10, 2.12)
Social interactions	Not meeting any friends	1	1
	Meeting some friends	2.06 (0.51, 8.23)	0.46 (0.26, 0.82)
**(d) Health conditions**			
Depressive symptoms	Normal	1	1
	Mild	0.79 (0.29, 2.19)	1.39 (0.81, 2.38)
	Moderate	1.14 (0.29, 4.50)	1.45 (0.65, 3.26)
	Severe	3.90 (1.13, 13.47)	1.91 (0.81, 4.50)
Activities of daily living	Independent	1	1
	Partially disabled	0.73 (0.18, 2.89)	2.44 (1.30, 4.56)
	Disabled	0.32 (0.04, 2.64)	2.97 (1.43, 6.14)

^*^Models considered pre-disaster baseline characteristics such as sex, age, education, number in household, social interactions, physical height (cm), body mass index, depressive symptoms, activities of daily living, comorbidity (cancer, heart diseases, stroke, respiratory diseases), and health behaviors (smoking, alcohol drinking, and exercise). ^**^On the day of the disaster, to reduce the possibility of biased estimation from maximum likelihood estimation due to relatively smaller numbers of mortality events (N = 33), we applied logistic regression analysis with penalized maximum likelihood estimation.

Although statistically non-significant, physical strength (taller body height served as proxy) tended to be protectively associated with mortality on the day of the disaster (compared to taller people, participants with a height of less than 150 cm had a 2.98 times increased mortality rate (95% CI: 0.75, 11.84), but men had higher mortality (OR = 2.74 (95% CI: 0.77, 9.68)) compared to women (Table 2b).

Those with greater social connectedness paradoxically appeared to have higher mortality risk on the day of the disaster (Table 2c). Those living with others had relatively higher risk of mortality than those living alone, although these estimates were not significant due to the small number of events. Although social interactions with friends and neighbors were not protectively associated with death on the day of the disaster, in the 3-year extended follow-up interval, the association between social interactions and mortality reverted back to a protective pattern, which has normally been observed in epidemiological studies (Table 2c).

Severe depressive symptoms pre-dating the disaster were associated with elevated risk of death on the day of the disaster (OR = 3.90 (95% CI: 1.13, 13.47), Table 2d), although participants with severe depressive symptoms lived, not only in coastal areas, but also in inland areas (Fig. 3b). The mortality rates among disabled participants were lower on the day of the disaster, but higher after the disaster.

## Discussion

On the day of the March 11, 2011 disaster, 92.4% of deaths were due to drowning^26^. This situation differed from that noted during previous inland earthquakes in which many people died due to the collapse of their homes^31,34,35^. In this study field, the earthquake occurred at 2:46 p.m., and the tsunami reached the Iwanuma coastline at 3:56 p.m.^36^. Although there was a 1-hour warning interval between the earthquake and the tsunami, some residents in the coastal areas still failed to evacuate. Our study examined pre-existing risk factors for mortality among older people. In addition to residential distance from the coastline, we found that presence of depressive symptoms before the disaster was a major risk factor for mortality on the day of the disaster. This new finding was made possible only as a result of our unique study design. In addition, people with stronger social relationships tended to be at increased risk of mortality on the day of the disaster, possibly because they tried to help others. However, in the months and years following the disaster, a protective impact of social interactions was observed. Other risks of mortality after the disaster were also primarily driven by conventional risk factors (such as older age). These findings contribute to deepening our understanding of the life-threatening impact of disasters.

The present study has several strengths. Explanatory variables were available pre-dating the disaster. This “natural experiment” design enabled us to avoid recall bias in the aftermath of the disaster, and to produce robust evidence for the creation of disaster preparedness policies.

This study also has several limitations. First, the response rate was only 59.0% at the baseline (pre-disaster) survey. However, as a whole, comparison to census data in Iwanuma supported the representativeness of the present data^37^, and the follow-up rate for the mortality outcome was high (99.6%). In addition, the response rate was comparable to that in other surveys involving community-dwelling residents^38^. For example, response rates of community-dwelling resident surveys in 10 European countries varied from 37.6% to 73.6%^38^. Second, although the GDS-15 has been validated as a measure of depressive symptoms, most of the variables in our survey were self-reported.

Some residents in the coastal areas failed to evacuate in spite of the 1-hour interval between the earthquake and the tsunami. There were several reasons for this. First, the earthquake damaged the electric power supply and impeded communications, which contributed to delays in evacuation. In addition, past tsunami experiences may have lulled some people into a false sense of security and they did not feel evacuation was necessary^39^. In the year prior to the disaster (February 28, 2010), the highest level tsunami alert was issued in the same area followed by evacuation of residents; however, the tsunami did not even reach the land on that occasion. In addition, two days prior to the March 11 event, a lower level tsunami alert was issued, but again the waves did not reach the land. These past tsunami experiences are believed to have contributed to a false sense of security among some local residents and delayed their evacuation. Third, there was a possibility that some heuristic-based biases distorted evacuation decision making^28^. Normality bias, which is the human’s propensity to consider abnormalities as normal, may have contributed to delaying evacuation^27^. This cognitive bias can be potentiated by groupthink^28^. These heuristic-based biases can delay evacuation, for example, waiting for others to be able to evacuate together. In the present study, people living with other family members tended to have higher mortality rates on the day of the disaster, even though social isolation is an established risk factor for death in normal situations^40,41^. In particular, people living with parents who were more vulnerable because of higher age^30–32^ had higher risk of mortality. This finding might be explained by pro-social behavior, i.e. helping others to evacuate, that may have increased the risk of being caught in the tsunami^27^. In the Indian Ocean tsunami, family members helping each other is also believed to have paradoxically decreased the chances of survival^22^. Therefore, higher mortality among men on the day of the disaster might also be explained by altruistic behavior. There is a possibility that an older adult with disability would be more likely to live with other family members. In fact, however, in the Japan Gerontological Evaluation Study (JAGES) cohort, people living alone tended to be older and to report lower activities of daily living (ADLs) compared to those co-habiting with others (Supplementary Table 4). In the analysis, we adjusted for pre-disaster disability status and other covariates. It is also possible that social connectedness was correlated with death due to delayed evacuation while waiting for others’ help to arrive.

A novel finding of the present study is that severe depressive symptoms also delayed evacuation ahead of the tsunami. There are possible explanations to account for the correlation between depressive symptoms and delayed evacuation. Delayed response is observed among depressed individuals because of the changes in brain function in the reward-related regions^42,43^. Because of decreased reward processing, it is difficult for depressed individuals to modify unhealthy behavior to improve health^44^. In addition, depressed older people tend to have cognitive impairment and dementia^45^. Psychomotor retardation, which includes motor and cognitive impairment, is a major feature of depression^46^. Feelings of hopelessness – i.e. negative expectation bias about the outcome of any attempt to save themselves - may have also reduced the motivation to evacuate^47^. These mechanisms may explain delayed evacuation among participants with severe depressive symptoms.

There are some possible reasons why distance from the coast, the proxy of personal experiences of disaster damage, did not have a clear effect on mortality after the disaster. In relocating the displaced survivors of the disaster, Iwanuma City officials consciously strove to maintain the social networks of residents. Most survivors were moved to shelters and temporary housing together with their neighbors; thus, even after the disaster, they successfully kept their “community” that existed before the disaster. Therefore, many survivors were able to maintain the same social networks as before the disaster and this seemed to be beneficial to their mental health^48,49^. The residences to house the displaced survivors were also built within one block of a large local hospital, so that geographical barriers to health care access were minimized.

There are also some notable similarities between the present study and previously reported findings from the Indian Ocean tsunami^22^. In both studies, living closer to the coast, shorter body height, and living with older people were associated with higher mortality, while socioeconomic status did not have a strong impact. In the Indonesia study, physical strength, which is related to swimming and running ability, was a key factor for survival during the tsunami (male sex and body height were the proxies for these characteristics), whereas being female, being a child or an older adult, having a shorter height, and living with an older woman were each associated with higher tsunami mortality^22^. Therefore, the present results relating to these risk factors could be generalized to other populations. In contrast, in our study, physical strength was not necessarily associated with survival, though shorter height and older age tended to be associated with heightened mortality risk. In fact, mortality risks were lower among women, the disabled, and those reporting less physical activity prior to the disaster. One possible reason for this is that older people living in nursing homes were assisted by the staff to evacuate in Iwanuma City^50^. In addition, the discrepant results between the Indian Ocean tsunami and the current study may be partly ascribed to differences in water temperature since Sumatra is located at a latitude of 5°N and Iwanuma City at a latitude of 38°N. The water temperature around the study area at the time was lower than 0 °C^51^. Cold-shock and hypothermia^52^ would have been major causes of mortality in the Japan tsunami, even if people, especially men, could swim.

Aldrich and Sawada^20^ conducted an ecological study of the determinants of mortality in the 2011 Great East Japan Earthquake and Tsunami. They reported that municipal levels of social capital – the lower level of local crime rate served as proxy – were associated with lower mortality. Because our study focused on individual mortality risk (as well as individual measures of social connectedness), we cannot directly compare our results to those of the previous study. Similar to our study, Weil, *et al*.^53^ reported longitudinal effects of social capital following exposure to disaster. Immediately following Hurricane Katrina in the U.S., people with higher social capital experienced increased stress because they helped displaced victims, but thereafter they were found to quickly recover.

Social connectedness was beneficial for longer term survival, which is consistent with previous studies^41^. Social connectedness is thought to reduce mortality among disaster survivors by acting as a buffer against stress^41^. Social support relating to altruistic behavior from neighbors could have helped to reduce emotional stress, and served as a source of instrumental assistance in daily life. Previous studies reported favorable association of social capital and recovery after disaster^53–58^. According to Aldrich^58^ the mechanisms by which local social capital enhance disaster recovery include mutual assistance between residents, collective action, and stronger linkage between victims and government services.

The present results include a public health implication: disaster evacuation planning should take into account that those with depressive symptoms have greater difficulty in evacuation situations than those who are not depressed. In addition, since it is difficult to behave as disaster experts wish when disaster occurs^59^, sharing understanding about human’s pro-social behavior and cognitive bias should be included in emergency public health education. Although our study examined the risks of tsunami-related mortality, further evidence is needed for other types of disasters, which may differ with respect to factors that predict survival.

In conclusion, this natural experiment study produced robust evidence for the creation of disaster preparedness policies. Pre-existing individual, social, and health characteristics affect mortality risk during and after a major disaster, and the effects of social connectedness may differ during versus after a disaster.

## Methods

### Cohort study design

**Figure 4:**
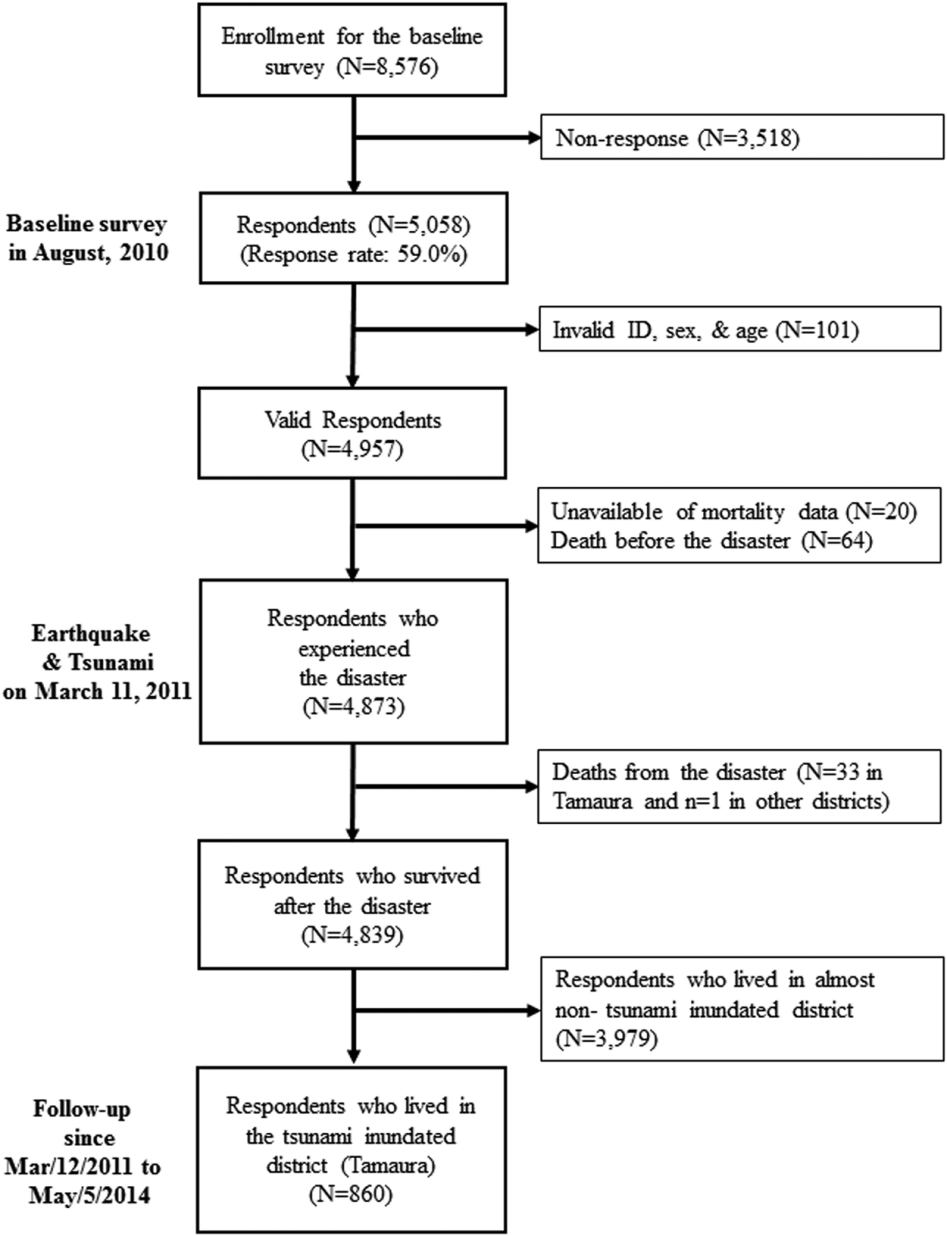
Japan Gerontological Evaluation Study/Iwanuma Project cohort composition.

This study is part of a larger ongoing, nationwide prospective cohort study, called the Japan Gerontological Evaluation Study (JAGES)^37,60–62^. In August 2010, a census of all residents aged 65 years or older was conducted in Iwanuma City, Miyagi Prefecture, for studying the determinants of healthy aging and functional disability. At that time, Iwanuma had a population of 44,187 living over an area of 60.71 km^2^. A census was taken of all the city’s residents aged over 65 years. Questionnaires were sent to 8,576 residents and 5,058 were returned. The response rate was 59.0%, which is comparable to other surveys of community-dwelling residents in European countries^38^. Seven months following the establishment of the cohort baseline, the east coast of Japan (including Iwanuma) was struck by a magnitude 9.0 earthquake and tsunami. On March 11, 2011, the earthquake occurred at 2:46 p.m., and the first wave of the tsunami reached the Sendai Airport (Fig. 1) located in Iwanuma at 3:56 p.m.^36^, though the time the tsunami arrived varied between municipalities. The height of the tsunami around the Sendai Airport was 5.6 to 12.3 m^63^. Figure 1 depicts the heat map of mortality in relation to the tsunami-inundated areas of Iwanuma city. The tsunami swept 48% of the municipality’s land area, and 187 residents including younger people lost their lives. This study is therefore a unique “natural experiment” in which we had information about the residents of a disaster-affected area that pre-dated the event. To determine the risk factors for mortality on the day of the disaster, we restricted our analysis to residents of Tamaura-district where all of the land area was inundated (N = 860). A flow chart of the participants is shown in Fig. 4. ArcGIS Pro version 1.1.1. (Esri, Redlands, CA) was used for drawing the map.

### Mortality assessment

Our outcome was all-cause mortality. Mortality data of the participants up to May 5, 2014 were obtained from the national long-term care insurance database. Local physicians are required to report all deaths to the local municipal government. The insurance database enabled us to link 99.6% of cohort participants.

### Social and demographic predictors

We measured each resident’s distance from the coast as an indicator of tsunami damage. We used years of educational attainment as an indicator of socioeconomic status. Social connectedness was measured by household living arrangement: living alone, living with parent(s), or co-habiting with others (e.g. spouse, adult children), but not parent(s), and by social interactions (frequency of informal socializing with friends-not meeting any friends or meeting some friends). If participants lived with parent(s) and others, they were categorized as “living with parent(s)”.

### Health status and health behavior

Comorbidity was determined by inquiring about current medical treatment for the following conditions related to major causes of death in Japan: cancer, heart disease, stroke, and respiratory diseases^64^. We also inquired about body height, since this had been previously reported to be associated with tsunami-related mortality^22,65^. Depressive symptoms were assessed by the Japanese version^66^ of the Geriatric Depression Scale (GDS-15)^67^. Total scores of GDS were categorized as follows: 0–4, normal; 5–8, mild; 9–11, moderate; and 12–15, severe^68^. ADLs were determined by asking whether participants could walk, take a bath, or use the toilet independently. We also inquired about the following health behaviors: smoking, alcohol consumption, and physical exercise (i.e. walking time per day in min). Body mass index (BMI) was also calculated from self-reported height and weight.

### Statistical analyses

The Kaplan-Meier survival curves from the day of the disaster up to three years of follow-up among all survey participants (N = 4,937) are shown in Fig. 2. The mortality rates on the day of the disaster according to distance of residents from the coastline (Tamaura district, N = 860) are shown in Fig. 3a. The prevalence of the participants with severe depressive symptoms at the baseline by distance living from the coast in Tamaura districts (N = 860) are shown in Fig. 3b.

We separately analyzed risk factors for mortality on the day of the disaster versus up to 38 months after the disaster. We applied logistic regression models to determine the risk of mortality on March 11, 2011, the day of the disaster. Because of the small number of events (N = 33 deaths) relative to the number of predictor variables, we applied logistic regression with penalized maximum likelihood estimation for rare events analysis^69^. Next we used Cox proportional hazards models to examine mortality risk from one day after the disaster up to 3.15 years (38 months) after the disaster (March 12, 2011 to May 5, 2014).

We applied the missing at random assumption, and created 10 datasets using the multivariate normal imputation method^70^. Results of univariate analyses, results of sensitivity analyses, and information on the missing responses are shown (Supplementary Table 1, 2, and 3). STATA SE version 14.1 (Stata Corp, College Station, TX) was used for all analyses.

### Ethics statement

The study was reviewed and approved by the Human Subjects Committee of the Harvard T. H. Chan School of Public Health, the Ethics Committee of the Tohoku University Graduate School of Medicine, the Research Ethics Committee of the Graduate School of Medicine, Chiba University, and the Research Ethics Committee involving Human Participants of the Nihon Fukushi University. Explanations of the study and the self-reported questionnaire were sent by mail to the residents. They were informed that participation was voluntary and that returning the self-administered questionnaire would be interpreted as implying consent.

We followed the STROBE Statement to report our observational study.

### Data availability statement

All data used are from the JAGES study. The JAGES data used in this study will be made available upon request, as per NIH data access policies. All enquiries are to be addressed to the data management committee via e-mail: dataadmin.ml@jages.net. All JAGES datasets have ethical or legal restrictions for public deposition due to inclusion of sensitive information from the human participants.

## Electronic supplementary material

Supplementary information
