# Supplementary material for: Risk of mortality during and after the 2011 Great East Japan Earthquake and Tsunami among older coastal residents
Source: Sci Rep. 2017 Nov 29;7:16591. doi: 10.1038/s41598-017-16636-3 (PMC5707380; doi:10.1038/s41598-017-16636-3)
Supplement: Supplementary file 1 — Supplementary information [file 41598_2017_16636_MOESM1_ESM.pdf]

Supplementary information

Risk of mortality during and after the 2011 Great East Japan Earthquake and Tsunami among older coastal residents

Jun Aida[1,2,*], Hiroyuki Hikichi[3], Yusuke Matsuyama[2], Yukihiro Sato[2], Toru Tsuboya[2], Takahiro Tabuchi[4], Shihoko Koyama[2], SV Subramanian[3], Katsunori Kondo[5], Ken Osaka[2,6], Ichiro Kawachi[3]

[1]Tohoku University Graduate School of Dentistry, Center for Epidemiology, Biostatistics and Clinical Research, 4-1 Seiryo-machi, Aoba-ku, Sendai, Miyagi 980-8575, Japan

[2]Tohoku University Graduate School of Dentistry, Department of International and Community Oral Health, 4-1 Seiryo-machi, Aoba-ku, Sendai, Miyagi 980-8575, Japan

[3]Harvard T.H. Chan School of Public Health, Department of Social and Behavioral Sciences, 677 Huntington Avenue, Boston, Massachusetts 02115, USA

[4]Center for Cancer Control and Statistics, Osaka Medical Center for Cancer and Cardiovascular Diseases, 3–3–1, Nakamichi, Higashinari-ku, Osaka 537-8511, Japan

[5]Chiba University, Center for Preventive Medical Sciences, 1-8-1, Inohana, Chuo-ku, Chiba-shi, Chiba 260-8670, Japan

[6]Tohoku University, International Research Institute for Disaster, 468-1, Aramaki, Aoba-ku, Sendai, Miyagi 980-0845, Japan

* Corresponding. j-aida@umin.ac.jp

Supplementary Table 1. Risk for mortality on the day of the Great East Japan Earthquake and Tsunami based on the univariate logistic regression models and multivariate adjusted logistic regression models with/without multiple imputation*.

		Univariate odds ratio and 95% confidence interval (multiple imputation)	Multivariate adjusted odds ratio and 95% confidence interval (with multiple imputation)	Multivariate adjusted odds ratio and 95% confidence interval (without multiple imputation)
Distance from the coast (ref: 2000-4100 m)	0-500 m	20.05 (5.16, 77.90)	22.66 (5.78, 88.84)	22.05 (5.76, 84.43)
	500-1000 m	19.12 (4.92, 74.23)	16.88 (4.33, 65.84)	18.03 (4.68, 69.50)
	1000-2000 m	3.84 (0.75, 19.65)	3.01 (0.56, 16.16)	3.29 (0.62, 17.48)
Sex (ref: Women)	Men	2.06 (1.03, 4.11)	2.74 (0.77, 9.68)	1.65 (0.46, 5.90)
Age (ref: 65-69 years)	70-74 years	1.91 (0.56, 6.45)	2.25 (0.60, 8.44)	2.70 (0.68, 10.73)
	75-79 years	3.98 (1.33, 11.92)	5.29 (1.47, 19.04)	5.31 (1.39, 20.38)
	80-84 years	1.67 (0.44, 6.27)	2.32 (0.54, 9.98)	2.22 (0.47, 10.41)
	≥85 years	3.44 (1.05, 11.32)	5.41 (1.26, 23.16)	4.98 (1.10, 22.53)
Education (ref: >12 years)	<10 years	1.16 (0.37, 3.65)	0.55 (0.14, 2.11)	0.52 (0.14, 1.97)
	10-12 years	0.58 (0.14, 2.33)	0.49 (0.10, 2.36)	0.44 (0.09, 2.22)
Household (ref: Living alone)	Co-habiting with others, but not parent(s)	1.76 (0.35, 8.77)	3.04 (0.47, 19.74)	7.20 (0.62, 83.94)
	Living with parent(s)	3.90 (0.66, 22.99)	6.67 (0.83, 53.71)	21.07 (1.40, 316.36)
Social interactions (ref: Not meeting any friends)	Meeting some friends	1.00 (0.32, 3.16)	2.06 (0.51, 8.23)	1.80 (0.43, 7.58)
Height (ref: ≥160 cm)	<150 cm	1.05 (0.44, 2.53)	2.98 (0.75, 11.84)	2.95 (0.75, 11.62)
	150-159 cm	0.93 (0.35, 2.48)	2.20 (0.67, 7.19)	2.54 (0.77, 8.42)
BMI (ref: 18.5-24.9 kg/m^2)	<18.5 kg/m^2	1.22 (0.25, 5.89)	1.38 (0.21, 8.94)	1.30 (0.17, 9.71)
	≥25.0 kg/m^2	1.16 (0.53, 2.54)	1.28 (0.52, 3.11)	1.18 (0.46, 3.02)
Depressive symptoms (ref: Normal)	Mild	1.09 (0.44, 2.67)	0.79 (0.29, 2.19)	0.89 (0.29, 2.76)
	Moderate	1.41 (0.45, 4.38)	1.14 (0.29, 4.50)	1.84 (0.44, 7.63)
	Severe	3.44 (1.25, 9.44)	3.90 (1.13, 13.47)	6.86 (1.73, 27.17)
ADL (ref: Independent)	Partially disabled	1.10 (0.35, 3.40)	0.73 (0.18, 2.89)	0.67 (0.15, 2.98)
	Disabled	0.79 (0.15, 4.18)	0.32 (0.04, 2.64)	0.35 (0.03, 4.58)
Comorbidity (ref: No)	Cancer	1.78 (0.47, 6.75)	1.80 (0.39, 8.23)	1.60 (0.34, 7.65)
Comorbidity (ref: No)	Heart diseases	0.93 (0.36, 2.38)	0.74 (0.25, 2.16)	0.85 (0.29, 2.52)
Comorbidity (ref: No)	Stroke	1.99 (0.52, 7.59)	2.11 (0.44, 10.17)	2.26 (0.45, 11.40)

Comorbidity (ref: No)	Respiratory diseases	2.39 (0.62, 9.18)	1.87 (0.41, 8.59)	2.15 (0.45, 10.37)
Smoking (ref: Never)	Past	1.94 (0.91, 4.14)	1.73 (0.56, 5.29)	2.57 (0.67, 9.80)
	Current	1.85 (0.68, 5.00)	2.60 (0.63, 10.69)	3.66 (0.82, 16.40)
Alcohol (ref: Drink)	Quitted	1.72 (0.49, 6.10)	0.89 (0.20, 4.01)	0.69 (0.13, 3.77)
	Non	0.92 (0.42, 2.00)	1.29 (0.49, 3.44)	1.43 (0.54, 3.81)
Exercise (ref: ≥90 minutes)	<30 minutes	0.76 (0.28, 2.07)	0.59 (0.19, 1.90)	0.53 (0.16, 1.77)
	30-59 minutes	0.65 (0.22, 1.94)	0.70 (0.21, 2.36)	0.66 (0.19, 2.27)
	60-89 minutes	0.62 (0.16, 2.43)	0.61 (0.13, 2.89)	0.57 (0.12, 2.74)

*On the day of the disaster, to reduce the possibility of biased estimation from maximum likelihood estimation due to relatively smaller numbers of mortality events (N=33), we applied logistic regression analysis with penalized maximum likelihood estimation.

Supplementary Table 2. Risk for mortality after the Great East Japan Earthquake and Tsunami based on the univariate Cox proportional hazard model and multivariate Cox proportional hazard model with/without multiple imputation.

		Univariate odds ratio and 95% confidence interval (multiple imputation)	Multivariate adjusted odds ratio and 95% confidence interval (with multiple imputation)	Multivariate adjusted odds ratio and 95% confidence interval (without multiple imputation)
Distance from the coast (ref: 2000-4100 m)	0-500 m	2.69 (1.67, 4.33)	0.84 (0.43, 1.68)	0.76 (0.38, 1.53)
	500-1000 m	0.96 (0.51, 1.83)	0.76 (0.38, 1.51)	0.79 (0.40, 1.57)
	1000-2000 m	0.87 (0.46, 1.61)	0.83 (0.42, 1.65)	0.87 (0.43, 1.75)
Sex (ref: Women)	Men	1.18 (0.78, 1.76)	3.33 (1.44, 7.73)	2.99 (1.41, 6.37)
Age (ref: 65-69 years)	70-74 years	3.83 (1.38, 10.63)	3.53 (1.24, 10.03)	3.46 (1.21, 9.90)
	75-79 years	5.10 (1.88, 13.83)	3.41 (1.19, 9.80)	3.17 (1.08, 9.32)
	80-84 years	6.40 (2.38, 17.25)	4.65 (1.61, 13.46)	4.46 (1.52, 13.12)
	≥85 years	21.48 (8.48, 54.36)	10.90 (3.82, 31.10)	10.00 (3.46, 28.90)
Education (ref: >12 years)	<10 years	2.84 (0.84, 9.58)	1.46 (0.42, 5.07)	1.53 (0.46, 5.08)
	10-12 years	3.49 (1.01, 12.06)	2.70 (0.75, 9.81)	2.89 (0.83, 10.02)
Household (ref: Living alone)	Co-habiting with others, but not parent(s)	0.38 (0.23, 0.61)	1.05 (0.54, 2.06)	0.83 (0.38, 1.81)
	Living with parent(s)	0.11 (0.03, 0.47)	0.45 (0.10, 2.12)	0.41 (0.08, 2.03)
Social interactions (ref: Not meeting any friends)	Meeting some friends	0.18 (0.12, 0.28)	0.46 (0.26, 0.82)	0.40 (0.22, 0.70)
Height (ref: ≥160 cm)	<150 cm	1.44 (0.86, 2.41)	1.31 (0.57, 2.97)	1.41 (0.61, 3.28)
	150-159 cm	0.96 (0.53, 1.76)	1.06 (0.52, 2.17)	1.06 (0.51, 2.19)
BMI (ref: 18.5-24.9 kg/m^2)	<18.5 kg/m^2	4.98 (2.83, 8.76)	2.07 (1.00, 4.27)	1.91 (0.90, 4.05)
	≥25.0 kg/m^2	0.75 (0.41, 1.36)	0.98 (0.50, 1.93)	1.01 (0.52, 1.93)
Depressive symptoms (ref: Normal)	Mild	2.08 (1.26, 3.43)	1.39 (0.81, 2.38)	1.51 (0.84, 2.74)
	Moderate	2.13 (1.05, 4.33)	1.45 (0.65, 3.26)	1.46 (0.66, 3.23)
	Severe	3.65 (1.82, 7.35)	1.91 (0.81, 4.50)	1.69 (0.70, 4.09)
ADL (ref: Independent)	Partially disabled	4.89 (2.94, 8.14)	2.44 (1.30, 4.56)	2.34 (1.24, 4.42)
	Disabled	10.07 (6.12, 16.55)	2.97 (1.43, 6.14)	2.59 (1.18, 5.67)
Comorbidity (ref: No)	Cancer	2.91 (1.51, 5.61)	1.21 (0.56, 2.62)	1.23 (0.53, 2.82)
Comorbidity (ref: No)	Heart diseases	1.21 (0.72, 2.01)	0.88 (0.48, 1.62)	0.85 (0.48, 1.52)
Comorbidity (ref: No)	Stroke	1.78 (0.78, 4.06)	0.59 (0.23, 1.53)	0.55 (0.21, 1.44)

Comorbidity (ref: No)	Respiratory diseases	2.80 (1.30, 6.05)	2.73 (1.06, 6.98)	2.95 (1.10, 7.89)
Smoking (ref: Never)	Past	1.01 (0.63, 1.63)	1.14 (0.57, 2.29)	1.42 (0.67, 2.97)
	Current	0.79 (0.39, 1.60)	0.92 (0.38, 2.26)	1.04 (0.42, 2.55)
Alcohol (ref: Drink)	Quitted	1.47 (0.49, 4.38)	0.77 (0.23, 2.56)	0.63 (0.18, 2.21)
	Non	2.06 (1.18, 3.59)	1.60 (0.79, 3.24)	1.59 (0.80, 3.16)
Exercise (ref: ≥90 minutes)	<30 minutes	8.10 (1.96, 33.39)	2.32 (0.52, 10.30)	2.41 (0.55, 10.46)
	30-59 minutes	3.90 (0.91, 16.84)	2.39 (0.53, 10.73)	2.16 (0.47, 9.87)
	60-89 minutes	3.58 (0.78, 16.39)	1.65 (0.34, 8.05)	1.84 (0.36, 9.29)

Supplementary Table 3. Number of missing responses and mortality

	On the day of the disaster (Mar/11/2011, N=860, N of mortality=33)	After the disaster (Mar/12/2011 to May/5/2014, N=827, N of mortality=95)
	Number of missing (mortality (%) for missing data)	Number of missing (mortality (%) for missing data)
Distance from the coast	0 (0)	0 (0)
Sex	0 (0)	0 (0)
Age	0 (0)	0 (0)
Education	98 (3.1)	95 (25.3)
Household	115 (4.4)	110 (16.4)
Social interactions	79 (10.1)	71 (16.9)
Physical height (cm)	115 (6.1)	108 (21.3)
BMI	123 (5.7)	116 (20.7)
GDS question 1	38 (5.3)	36 (19.4)
GDS question 2	47 (8.5)	43 (20.9)
GDS question 3	51 (3.9)	49 (20.4)
GDS question 4	58 (8.6)	53 (15.1)
GDS question 5	43 (4.7)	41 (14.6)
GDS question 6	48 (4.2)	46 (17.4)
GDS question 7	37 (5.4)	35 (20.0)
GDS question 8	49 (4.1)	47 (19.2)
GDS question 9	43 (2.3)	42 (21.4)
GDS question 10	41 (4.9)	39 (20.5)
GDS question 11	46 (8.7)	42 (16.7)
GDS question 12	39 (5.1)	37 (21.6)
GDS question 13	54 (5.6)	51 (19.6)
GDS question 14	58 (5.2)	55 (12.7)
GDS question 15	61 (8.2)	56 (10.7)
ADL	28 (3.6)	27 (7.4)
Comorbidity: Cancer	36 (2.8)	35 (5.7)
Comorbidity: Heart diseases	36 (2.8)	35 (5.7)
Comorbidity: Stroke	36 (2.8)	35 (5.7)
Comorbidity: Respiratory diseases	36 (2.8)	35 (5.7)
Smoking	108 (1.9)	106 (10.4)
Alcohol	36 (2.8)	35 (14.3)
Exercise	94 (3.2)	91 (28.6)

Supplementary Table 4. Cross-tabulation (without multiple imputation) and correlation of household on age, ADL, and socioeconomic status (SES).

Household	N	Age (%) 65-69 years	70-74 years	75-79 years	80-84 years	≥85 years	ADL (%) Independent	Partially disabled	Disabled	SES (education, %) <10 years	10-12 years	>12 years
Living alone	68	17.6	14.7	17.6	13.2	36.8	47.1	22.1	29.4	50.0	14.7	8.8
Co-habiting with others, but not parent(s)	618	29.6	22.7	20.4	15.5	11.8	88.3	7.1	2.8	62.0	24.9	9.4
Living with parent(s)	59	47.5	22.0	13.6	10.2	6.8	86.4	6.8	0.0	52.5	23.7	18.6
Spearman's correlation		-0.012 (P=0.724)					-0.040 (P=0.242)			0.024 (P=0.119)		
